# Resolving anaphoras for the extraction of drug-drug interactions in pharmacological documents

**DOI:** 10.1186/1471-2105-11-S2-S1

**Published:** 2010-04-16

**Authors:** Isabel Segura-Bedmar, Mario Crespo, César de Pablo-Sánchez, Paloma Martínez

**Affiliations:** 1 Computer Science Department, University Carlos III of Madrid, Leganés, 28921, Spain; 2 Biomedical Engineeering and Telemedicine Lab, University of Cadiz, Cádiz, 11002, Spain

## Abstract

**Background:**

Drug-drug interactions are frequently reported in the increasing amount of biomedical literature. Information Extraction (IE) techniques have been devised as a useful instrument to manage this knowledge. Nevertheless, IE at the sentence level has a limited effect because of the frequent references to previous entities in the discourse, a phenomenon known as 'anaphora'. DrugNerAR, a drug anaphora resolution system is presented to address the problem of co-referring expressions in pharmacological literature. This development is part of a larger and innovative study about automatic drug-drug interaction extraction.

**Methods:**

The system uses a set of linguistic rules drawn by Centering Theory over the analysis provided by a biomedical syntactic parser. Semantic information provided by the Unified Medical Language System (UMLS) is also integrated in order to improve the recognition and the resolution of nominal drug anaphors. Besides, a corpus has been developed in order to analyze the phenomena and evaluate the current approach. Each possible case of anaphoric expression was looked into to determine the most effective way of resolution.

**Results:**

An F-score of 0.76 in anaphora resolution was achieved, outperforming significantly the baseline by almost 73%. This ad-hoc reference line was developed to check the results as there is no previous work on anaphora resolution in pharmalogical documents. The obtained results resemble those found in related-semantic domains.

**Conclusions:**

The present approach shows very promising results in the challenge of accounting for anaphoric expressions in pharmacological texts. DrugNerAr obtains similar results to other approaches dealing with anaphora resolution in the biomedical domain, but, unlike these approaches, it focuses on documents reflecting drug interactions. The Centering Theory has proved being effective at the selection of antecedents in anaphora resolution. A key component in the success of this framework is the analysis provided by the MMTx program and the DrugNer system that allows to deal with the complexity of the pharmacological language. It is expected that the positive results of the resolver increases performance of our future drug-drug interaction extraction system.

## Background

A drug-drug interaction occurs when one drug influences the level or activity of another drug. Drug-drug interactions are common adverse drug reactions and unfortunately they are a frequent cause of death in hospitals [1]. Several published drug safety issues have showed that adverse effects of drugs may be detected too late, when millions of patients have already been exposed [2]. Therefore, they have an important impact on patient safety because they can be quite dangerous and their relatively high incidence among certain population groups such as geriatric or polydrug patients. In addition, drug interactions account for 16.6% of adverse drug reactions causing hospitalization [3], thus they are a direct cause of the increase of health care costs.

There are different resources which describe information about drug interactions (for example, DRUG-REAX System or the drug interaction appendix of the British National Formulary, but unfortunately there is a lack of consistency in the inclusion and grading of drug interactions across them [4], and they rarely include the whole range of drug interactions reported in the medical literature [5]. Therefore, the development of automatic methods for collecting, maintaining and interpreting this information is crucial to achieve a real improvement in their early detection. Natural Language Processing can provide an interesting way to reduce the time spent by health care professionals on reviewing the literature.

**Figure 1:**
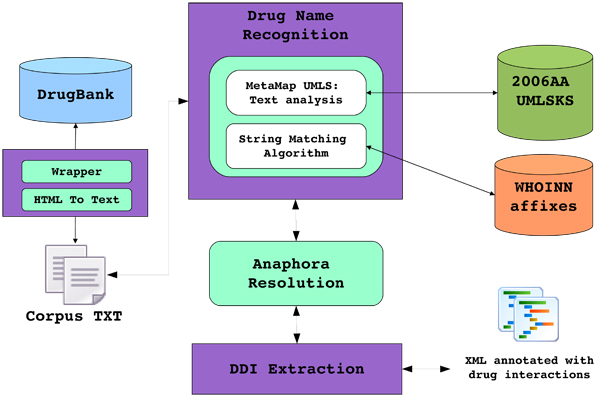
**Architecture for drug-drug interactions extraction** This figure shows the pipeline architecture of our drug-drug interaction prototype. Firstly, texts are processed by the MMTx program. This tool performs sentence splitting, tokenization, POS-tagging, chunking, and linking of phrases with UMLS concepts. Then, the drugs found in such documents are classified into drug families by a set of nomenclature rules (WHOINN affixes) recommended by the World Health Organization (WHO) International Nonproprietary Names (INNs) Program to identify and classify pharmaceutical substances. Over this basis, anaphora resolution is carried out to account for both nominal phrases referring to drugs and pronouns. Finally, the output of the previous modules is sent to the relation extraction module that exploits this information in order to account for drug interactions in biomedical documents.

This proposal is included in the broader context of an automatic system to extract drug interactions from pharmacological texts (see Figure 1). Drug-Drug Interaction Extraction is a difficult task whose complexity increases when one or both drugs involved in an interaction are expressed with an anaphoric expression, as shown in the following text excerpts taken from the DrugBank database [6,7]:

1. Although **beta-adrenergic blockers** or **calcium channel blockers** and **digoxin** may be useful in combination to control atrial fibrillation, **their additive effects** on AV node conduction can result in advanced or complete heart block.

2. In addition** triamterene, metformin** and **amiloride** should be co-administered with care as **they** might increase dofetilide levels.

Anaphora resolution is often a task required to improve the results of automatic extraction systems. Anaphoric relations can be found within the sentence level or even among different senteces. Although approaches to anaphora resolution in the literature vary in the use of features and in the accounted scope between the anaphoric expression and its antecedent, they can be grouped into two major approaches:

1. Heuristic approaches that integrate different knowledge sources like gender and number agreement, syntactic patterns or semantic information to obtain a plausible list of candidates [8-10]. The major drawback of these approaches is that it is very labor-intensive and time-consuming to construct the domain knowledge base necessary for resolving the anaphors.

2. Machine learning approaches compute the most likely candidate based on previous examples. These approaches can sort out the referred problem in heuristic approaches, however it usually comes across the data sparseness problem of language modeling, so they require a large amount of data to train [11,12].

In the biomedical domain, the lack of available corpora motivated that early approaches were mostly based on heuristics. In this sense, Castano et al. [13] present a method for resolving anaphoric expressions for candidates taken from MedLine articles and abstracts. By defining a different range of resolution scope for each type of anaphoric expression, it uses different morphological, syntactic and semantic features such as number or semantic type agreement (UMLS typing-based system), longest common subsequence for similarity among candidate antecedents and coercion-type matching (most suitable agent / patient linguistic role according to the verb) from the most frequent bio-relevant verbs in Medline. Each possible antecedent of a certain anaphora was given a different cumulative score according to the significance of its linguistic features and the one with the best salience measure was chosen. General results are 73.8% F-score over a corpus of 46 MedLine abstracts which were annotated by a domain expert.

Lin et al. [14] also apply this scoring technique but they restrict the types of nominal anaphoric expressions to be taken into account, enrich the syntactic features with new values and apply coercion-type matching as before, using Genia corpus [15]. General results are 92% F-score in pronominal anaphora and 78% in nominal anaphora in 32 Medline abstracts (MedStract) [16]. This approach is improved in [17] by using new resources like WordNet or PubMed for finding semantic relationships among concepts not found in UMLS. They extend the MedStract corpus with 100 Medline abstracts obtaining 87.43% F-score for pronominal anaphora and 80.61% for nominal anaphora.

Anaphora resolution applied to the field of protein interactions can be found in [18], which presents an anaphora resolution system integrated in a larger protein-protein interaction extraction study, so-called BioAR. It identifies antecedents of pronouns by applying patterns for parallelism and centering theory [19]. Nominal phrase anaphors are identified according to the most salient score, using similar features as in [13,20]. Experimental results are 75% precision and 56.3% recall in pronoun resolution and 75% precision and 52.2% in definite noun phrase resolution from 120 unseen biological interactions extracted by BiolE system [20].

Likewise, in [21] the impact of anaphora resolution on the result of a protein interaction extraction system is analyzed by using the Guitar system [22] over the 20 full texts and abstracts of the Medstract corpus and three articles taken from the Journal of Biological Chemistry. From the 402 protein-protein interactions in the corpus, only 20 were conveyed by an anaphoric expression. Results show 70% recall in anaphora resolution in abstracts and 52.65% in full texts. No data about precision are available. Results suggest small improvements in protein extraction.

Regarding machine learning approaches to anaphora resolution in biomedical documents, Nguyen and Kim [23] carries out a comparative study with three different corpora: MUC and ACE, accounting for the news domain, and Genia for bio-medical documents. They build a machine learning-based pronoun resolver using a Maximum Entropy ranker model that selects the most likely antecedent candidate from a set of candidates by using a huge set of linguistic features divided into baseline attributes like pronoun type, number, gender, string, distance, etc.(mostly used in other approaches) and innovative features like grammatical roles, most semantically appropriate candidate or context information about the anaphoric pronoun. From the latter group, those improving baseline for each of the corpus were selected obtaining 79.55% (Genia), 64.61%(ACE), 60.42%(MUC) in success rate.

Anaphoric expressions are resolved in [24] presenting a semisupervised approach that makes use of rich domain resources such as the FlyBase database. Nominal phrases are identified by the use of the domain-independent parser RASP [25]. The system was evaluated against two hand-annotated full papers containing 302 sentences and 314 anaphoric expressions. It looks for the closest antecedent matching the anaphoric expression according to a set of linguistic features. System reaches 58.8% precision and 57.3% recall. A summary of the main approaches of biomedical anaphora resolution can be found in Table 1. The approach presented in this paper, DrugNerAR, works on drug-drug interaction documents following an heuristic approach for anaphora resolution partially motivated by the lack of a large annotated corpus in this domain. The range and order in the anaphora-antecedent matching is adopted from the model of Centering Theory [19]. Linguistic analysis is provided by the MMTx tool as proposed by [26] in which we developed an approach for anaphora resolution for drug-drug interactions documents based on a scoring method similar to other works in the biomedical domain [13,14,18]. Results show how this new approach outperforms [26] and offers an interesting possibility to be developed for other sub-domains in biomedicine.

**Table 1:** Summary of the main approaches to biomedical anaphora resolution

Authors	Approach	Corpus	Results
Castano et al. [13]	Scoring method	46 medline abstract	F=0.74
Lin et al. [14]	Scoring method	32 MedLine abstract (MedStract)	F=0.92 pronominal, F=0.78 nominal
Kim et al. [18]	Centering theory for pronominal anaphors and scoring method for nominal anaphors	120 biological interactions	F=0.64 pronominal, F=0.59 nominal
Liang and Lin [17]	Scoring method	MedStract + 100 Med- Line abstract	F=0.87 pronominal, F=0.80 nominal
Segura-Bedmar et al., [26]	Scoring method and a set of semantic and morphological restrictions	49 MedLine abstracts	F=0.85 pronominal, F=0.50 nominal
Nguyen and Kim [23]	Maximum Entropy ranker model	Genia	Success rate: 79.55%

## Methods

This section describes our approach for anaphora resolution in Drug-Drug Interaction documents. Figure 1 shows the pipeline architecture of our drug-drug interaction prototype. Firstly, texts are processed by the MMTx program. This tool performs sentence splitting, tokenization, POS-tagging, chunking, and linking of phrases with UMLS concepts. This way, MMTx allows to recognize a variety of biomedical entities occurring in texts. Then, drugs found in such documents are classified into drug families by the DrugNer system [27], which is is based on a set of nomenclature rules recommended by the World Health Organization (WHO) International Nonproprietary Names (INNs) [28] Program to identify and classify pharmaceutical substances. Over this basis, anaphora resolution is carried out to account for both nominal phrases referring to drugs and pronouns. Finally, the output of the previous modules will be sent to the relation extraction module that will exploit this information in order to account for drug interactions in biomedical documents.

### Corpus for drug anaphora resolution

Two different stages have been distinguished in the creation of this corpus: compilation and annotation.

#### Compiling and preprocessing the corpus

There is no corpus dedicated to the resolution of the anaphoric expressions occurring in drug interaction descriptions in pharmacological documents, so the first challenge was to build a corpus for research purposes.

DrugBank is an annotated database with about 4900 drug entries. Each entry contains more than 100 data fields that gather detailed chemical and pharmacological information (type, category, brand names, chemical formula, drug interactions, etc).

A collection of 49 unstructured and plain documents was taken randomly from the field 'interactions' in the DrugBank database. Documents have on average 40 sentences and 716 words. Documents were downloaded by using an automatic robot developed with the free tool openKapow [29].

**Figure 2:**
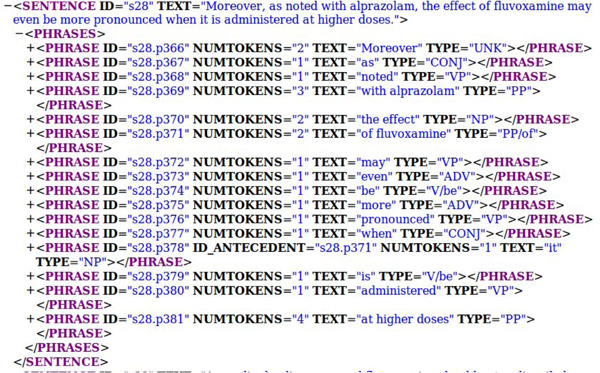
**Example of sentence processed by MMTx and DrugNer and annotated with anaphoric expressions** This example contains a pronominal anaphoric expression (phrase s28.p378,* 'it' *) whose antecedent is annotated by the attribute* ID-ANTENCENT.* In this case, the antecedent is the phrase s28.p371 (*'of fluvoxamine' *).

Each document was subsequently preprocessed by MMTx and the DrugNer system. Figure 2 shows an example of a preprocessed document. This example is limited to one input sentence without including information about tokenization. For each phrase, it is offered its type as well as the CUI, the name and the semantic types of the UMLS concepts provided by MMTx (just in case, the text of the phrase were founded in the UMLS Metathesaurus). Let us take as example the prepositional phrase s28.p369 (*'with aprazolam' *) which was mapped to the UMLS concept 'Alprazolam' (CUI='C0002333') whose semantic types are 'orch' (Organic Chemical) and 'phsu' (Pharmacological Substances). Moreover, the affix '-azolam' definited by WHOINN program allows to classify this phrase as a 'Benzodiazepine derivative'.

#### Annotating the corpus

Anaphora is a linguistic device to refer to entities that have come up in recent discourse (antecedents). There are two kinds of anaphors prevalent in this kind of literature:

• Pronominal anaphora. In this case an entity is referred to by a pronoun: personal (*it,they*), reflexive (*itself,themselves*), relative (*which, that*) and distributive (*both, each, either* and* neither*). Pronominal forms in first and second person (*I*, *me*, *you*, *your* and* who*) were disregarded for not referring to drugs.

• Nominal (phrase) anaphora. This is the case of an entity being referred to by a nominal phrase. These phrases consists of a definite article (*the*), possessive (*its, their*), demonstrative (*this, these, those*), distributive (*both, such, each, either, neither*) followed by a generic term for drugs (such as *antibiotic, medicine, medication,* etc) or a drug property or effect, e.g.,* these anticoagulants, its pharmacological effects.*

The corpus was annotated manually by a linguist with the assistance of a pharmaceutical expert over the output of MMTx and DrugNer. The example shown in Figure 2 also contains a pronominal anaphoric expression (phrase s28.p378,* 'it' *) whose antecedent is annotated by the attribute* ID-ANTENCENT.* In this case, the antecedent is the phrase s28.p371 (of fluvoxamine). The corpus contains a total of 331 anaphoric expressions (see tables 2 and 3). A more detailed description of the corpus can be found in [26].

**Table 2:** Distribution of pronominal anaphors in the corpus

Pronominal Anaphors	Num
Personal (it, they)	23
Reflexive (itself, themselves)	1
Relative (which, that)	113
Distributive (both each, either, neither	8
Demonstrative (these, this, those, that)	12
Indefinite (all, some, many, one)	8
Total Phrases:	165

**Table 3:** Distribution of nominal anaphors in the corpus.

Nominal Anaphors	Num
Definite (the)	37
Possessive (its, theirs)	52
Distributive (both, each, either, neither)	11
Demonstrative (these, this, those, that)	58
Indefinite (other, another, all)	8
Total Phrases:	166

### Linguistic rules-based method for drug anaphora resolution

The anaphora resolution issue can be split into three different phases: identification of anaphoric expressions, determination of anaphor scope and selection of antecedents.

#### Identification of anaphoric expressions

All pronouns referred to in table 2 for pronominal anaphora were selected. Moreover, the pleonastic-it expressions were excluded by using the rules proposed in [14]. These rules were extended to recognize the negation and modal verbs as possible arguments in this kind of expressions (see Table 4).

**Table 4:** Rules to recognize pleonastic-it expressions.

Rules	Examples
IT [MODALVERB [NOT]?]? BE [NOT]? [AJD|ADV| VP]* [THAT|WHETHER]	**It is not known whether** other progestational contraceptives are adequate methods of contraception during acitretin therapy.
IT [MODALVERB [NOT]?]? BE [NOT]? ADJ [FOR np] TO VP	If** it is not possible to discontinue** the diuretic, the starting dose of trandolapril should be reduced.
IT [MODALVERB [NOT]]? [SEEM|APPEAR|MEAN|FOLLOW] [THAT] *	**It does not appear that** the SSRIs reduce the effectiveness of a mood stabilizer in these populations

Regarding nominal phrase anaphora, candidates were selected if attached to a drug family (analgesics, anticoagulants, etc) or to a generic term for drugs (such as 'medicine', 'medication' or 'drug'). Candidates consisting of specific terms for drugs like 'aspirin', 'fluvoxamine', etc., were disregarded. To achieve this, our module uses the concept unique identifier (CUI) provided by MMTx to distinguish between generic and proper noun for drugs.

Candidate anaphors consisting of a possessive article were restricted by only selecting those phrases attached to the semantic type 'Qualitative Concept' in UMLS, that is, those accounting for drug properties or effects, e.g., 'its pharmacological effect', 'their anticoagulant properties'.

Finally for distinguishing nominal phrases and pronouns consisting of units 'both', 'either', 'neither' from correlative expressions, a regular expression (see Table 5) was developed.

**Table 5:** Regular expressions to detect correlative expressions.

Rule	Example
[BOTH|EITHER|NEITHER] [N P|P P|U NK] [AND|OR|NOR] [NP|PP|UNK]	These pharmacokinetic effects seen during diltiazem coadministration can result in increased clinical effects (e.g., prolonged sod ation)of **both midazolam and triazolam.**

Once a nominal candidate has been selected, it is necessary to determine its grammatical number. Unfortunately, MMTx does not provide this information, so every phrase's head noun was matched against a set of lexical patterns (see Table 6) to decide its number.

**Table 6:** Lexical patterns to determine grammatical number.

Number	Lexical pattern
Plural:	[A-Z]+(S|IES|OES|XES|SHES|CHES|SES|ZES)
Exception for singular:	[A-Z]+(U|S)S

Moreover, a regular expression is applied to detect coordinative structures occurring inside a sentence. This expression is helpful to resolve those anaphors matching plural antecedents if they are expressed by mean of a coordinative structures as shown in Table 7.

**Table 7:** Rules to detect coordinative structures.

Rule	Example
( [NP|PP|UNK],)* [NP|PP|UNK] [AND|OR|NOR] [NP|PP|UNK]	While all **the selective serotonin reuptake inhibitors ( SSRIs ) e.g. fluoxetine, sertraline and paroxetine** inhibit P450 2D6, they may vary in the extent of inhibition.

#### Determination of anaphora scope and antecedent selection

The range of searching for a possible antecedent is not unlimited. As referred, this approach makes use of the framework called 'centering' [19] to account for the way information is structured and focused linguistically. Entities (centers) referred to in an utterance serve to link that utterance to others in the segment that contains them. The main claims of this theory applied to anaphora resolution are the following:

1. The choice of a center (antecedent) for a certain anaphora is from the set of entities (centers) of the previous utterance (locality).

2. Entities mentioned in an utterance are more central than others according to this function (subject>object>other).

3. Each anaphoric expression in an utterance has exactly one antecedent (center).

In this approach, anaphoric expressions were associated to just one antecedent (third claim). This antecedent is taken from the previous ordered sequence of entities (centers) (first and second claims). Basically the system tries to match an anaphoric expression against candidates in the same sentence sorted by position from left to right. The more central an entity is, the higher the possibility it is to be located on the left side of a sentence (subjects are usually at the beginning); in case no antecedent matches, it moves backward up to the previous sentence and searches for antecedents from left to right again.

However, it was observed that the Centering Theory cannot account for certain types of anaphoric expressions whose antecedents are in most cases to be found locally. Relative, reflexive and possessive anaphoric expressions find their antecedent in the previous context in most of the cases, so it was decided not to apply Centering Theory on this kind of expressions and link them to the closest nominal phrase that satisfied their semantic and morphological restrictions.

For each of the ordered list of candidates selected in the previous phase, the system checks one by one whether their linguistic features are consistent with features of the anaphoric expression. Nominal phrases and pronouns present number agreement with their antecedents. Nominal phrases in coordinative or appositive relation were taken as the same center (antecedent). Additionally, nominal phrase anaphors following centering restrictions were determined to match nominal phrases representing drugs, in particular those phrases classified by MMTx according to one of the following semantic types: pharmacological substances (phsu), antibiotics (antb) or clinical drug (clnd). Likewise, these phrases must not be composed of abstract drugs (drug families or phrases such as 'the medicine' or 'this drug'), but a drug specifically.

## Results

As there is no previous work on anaphora resolution in pharmacological texts, it was decided to develop an ad-hoc baseline strategy for anaphora resolution that simply selects the closest nominal phrase. The anaphoric expressions considered are those referred to in subsection 'Annotating the corpus'. Regarding the present approach, results of the anaphora resolver were compared with those provided by the corpus. From the 331 anaphoric expressions considered, 265 were detected by the system and 232 were successfully attached to an antecedent. For testing system accuracy an F-score measure with *β *=1, also called a balanced F-score, a weighted harmonic mean of precision and recall were used. Global results of both baseline and present approach are shown in Table 8. Results for the different types of anaphora are shown in the tables 9 and 10.

**Table 8:** Global results for the baseline and the approach.

	Baseline			Centering Approach			
			
Total	Precision	Recall	F-baseline	Precision	Recall	F-approach	Inc
331	0.49	0.40	0.44	0.84	0.7	0.76	0.73

Increment (Inc) is defined as follows

**Table 9:** Results for pronominal anaphora resolution.

		Baseline			Approach			
				
Type	Total	P	R	F	P	R	F	Inc
Personal	23	0.26	0.26	0.26	0.91	1	0.95	2.65
Reflexive	1	1	1	1	1	1	1	0
Relative	120	0.83	0.81	0.82	1	0.99	0.99	0.21
Distributive	8	0.33	0.12	0.18	0.85	0.87	0.86	3.78
Demonstrative	11	0	0	0	0.33	0.27	0.29	∞
Indefinite	8	0.25	0.12	0.16	0.57	0.62	0.59	2.69
Global results	164	0.67	0.65	0.66	0.92	0.904	0.91	0.38

**Table 10:** Results for nominal anaphora resolution.

		Baseline			Approach			
				
Type	Total	P	R	F	P	R	F	Inc
Definite	37	0	0	0	0.54	0.59	0.56	TO
Possessive	52	0.53	0.42	0.47	0.76	1	0.86	0.83
Distributive	11	0.20	0.27	0.23	0.77	0.90	0.82	2.57
Demonstrative	58	0.03	0.01	0.02	0.81	0.48	0.60	29
Indefinite	8	0	0	0	0.40	0.37	0.38	to
Global results	166	0.23	0.15	0.18	0.71	0.47	0.56	2.11

The results obtained by DrugNerAR achieved an increment of 73% respect to the baseline and the system outperforms our previous approach for drug anaphora resolution based on constraints and scoring [26]. This is explicable since previous approach enphasized the proximity of the candidate to the anaphoric expression and antecedents can be found at the beginning of the same or previous sentence as it is pointed out by [19]. Regarding other approaches, our results are not directly comparable to these works, but partially:

1. Syntax changes from a domain to another. Most approaches in the biomedical domain deal with documents from MedLine accounting for any biomedical topic, whereas our documents focus on drug interactions. Subsequently, we consider that language style of our documents must be linguistically oriented to the reflection of such relations. Only works [18] and [21] deal with documents accounting for protein interactions.

2. Other works mostly address the anaphora resolution issue by using a set of morphosyntactic properties, so resolution is going to be determined by the way that a document has been analyzed. For example, expressions like* these drugs* or* this medication* are required to be analyzed by a knowledge resource that identifies and analyze them both syntactically (they are nominal phrases in the subject, object or other type of position in the sentence) and semantically (they stand for drugs). Some approaches make use of open-domain analyzers like [24] with RASP. Conversely, other approaches makes use of the corpus Genia that has been manually tagged and it does not contain annotation errors (this has a definite influence over results). The degree of precision in annotation is extremely important since results depend on such results. Our system makes used of MMTx, that although has shown to be useful for the analysis of biomedical texts, has several syntactic and semantic parsing errors.

To our opinion, from the list of approaches referred to in the Background section [18] is the closest to ours. As discussed, such an approach addresses the issue of anaphora resolution in the domain of protein interactions, has developed an ad-hoc tool called BioIE to deal with morphosyntactic complexity of this kind of documents and resolution problems have been faced with an approach that also used Centering Theory. As it can be seen in Table 1, our work obtains similar results to [18] for nominal phrase anaphora resolution and better results for pronominal anaphora.

## Conclusions

Compiling a comprehensive database of drug-drug interactions is a relation extraction task that requires the resolution of anaphoric expressions in biomedical and pharmacological texts. It is believed that anaphora resolution would improve the recall of any extraction method and it would be particularly useful for semiautomated compilation of drug-drug interactions.

The described approach for anaphora resolution uses Centering Theory in order to select the scope of the anaphoric expressions and assign the correct antecedent. In contrast, a simple heuristic that selects the closer nominal phrase has been experimentally useful in this domain for some types of expressions, relative pronouns and possessive nominal anaphors.

A key component of the approach is the use of several domain resources, including the MMTx biomedical parser and the UMLS meta-thesaurus. Other approaches that have deal with biomedical documents have used domain-independent parsers that do not adequately handle the syntactic complexity of biomedical language, including terminology. Unfortunately, MMTx only provides shallow syntactic information, so it can be expected that full syntactic parsing improves the performance of the linguistic rule-based analyzer. UMLS has been useful in order to identify the anaphors and implement semantic restrictions to candidate resolution.

Future work will consider the overall contribution of the anaphora resolution module in the broader task of drug-drug interaction extraction and their evaluation on a larger corpus. Although sources of interaction information like Medline abstracts and DrugBank may share a common literary style, the distribution of interactions is very different and it also deserves investigation. Moreover, semantic information about drug families provided by DrugNer can be valuable for the improvement in the resolution of certain nominal anaphors. In the following example, DrugNer could identify 'venlafaxine' like a antidepressant drug, and this would help to correctly resolve the anaphor 'the antidepressant effect', in the following sentence: *Coadministration of naloxone with **venlafaxine** did not modify **the antidepressant effect.***

Additional extensions of this work include the extending the coverage of the approach to other kinds of biomedical entities (such as genes, diseases or drug targets), the increasing of the size of the corpus in order to make more reliable conclusions, and the application of machine learning techniques that have been successfully applied on other domains.

## Competing interests

The authors declare that they have no competing interests.

## Authors contributions

IS developed the design and implementation of the system and participated in its evaluation. MC provided the annotation of the corpus and the design of the linguistic rules, and participated in the study of the related work. CP designed the arquitecture of the system, and took part in the implementation and evaluation of the system. PM carried out the study of the related work, participated in the design and coordination of work, and helped draft the manuscript. All authors read and approved the final manuscript.
